# 

**Published:** 1920-10-02

**Authors:** 


Supplement to "The Hospital," April 16, 1921.

### THE HOSPITAL

*THE MODERN NEWSPAPER OF ADMINISTRATIVE MEDICINE AND INSTITUTIONAL LIFE*

INCLUDING

ADMINISTRATION, NATIONAL INSURANCE, HEALTH

AND

THE INTERESTS OF ALL INSTITUTIONAL WORKERS

OCTOBER 1920 TO MARCH 1921

VOLUME LXIX

LONDON

PUBLISHED BY THE SCIENTIFIC PRESS (LIMITED), AT *THE HOSPITAL* BUILDING

28 & 29 SOUTHAMPTON STREET, STRAND, W.C. 2.

MDCCCCXXI

19364

41135A

### INDEX TO VOL. LXIX.

Abergavenny Workhouse, 16
Aberystwyth Board of Guardians, 308
Accommodation, Provision of Hospital, 407, 449, 469
Adopt a Hospital! 577
Advertisement, The Uses of, 237
Aeroplane and Health, The, 307
Agitation, On, 294
Alcohol, 8
Alexandra Day, 24
Alien Health Precautions, 573
Almoners: Views of the Almoners' Council, 131; Hospital Officers' Association, 301; The Almoner Question, 551, 592
Ambulance Work, Lay Service In, 182; Service in London, 527
American Nurse-Training Schools, 17
American Red Cross, 40, 108
American Society in London, The, 200
Amœbic Dysentery in Great Britain, 513
Anæsthetics, Deaths Under, 234
Anatomy Acts, The, 24, 140
Annual Reports, 556
Anthrax Danger, The, 424
Anti-Tubercle Serum (*see* Tuberculosis)
Anti-Typhus Appeal, 366
**APPOINTMENTS, MEDICAL:** 302, Jan. 1, iv, Jan. 15, vi, 468
**APPOINTMENTS: MATRONS AND SISTERS,** Oct. 2, x, 42, Oct. 16, vi, Oct. 23, vi, Oct. 30, vi, Nov. 6, viii, 158, 184, Nov. 27, x, Dec. 4, viii, 225, 250, 274, 302, Jan. 1, iv, 350, Jan. 15, vi, 398, Feb. 5 Supp., 2, 418, Feb. 12, viii, Feb. 18, vi, Feb. 26, vi, 528, 574, March 12, viii, 600
Are Nurses Necessary? 148
Armistice Day, 135, 161
Army Nurses in India, 85
Art of Keeping Clean, The, 564
Association for Moral and Social Hygiene, 31
Association of British Chemical Manufacturers, 487
Association of Hospital Matrons, 182
Astor, Lady, 595
Atmospheric Pollution, 187
Australasian Medical Conference, The, 78
Australia: Indigenous Animals, 118; Scientific Research, 118

Baby Week (*see* Infant Welfare)
Bagley, Miss Louisa, 543
Baker, Mr. William, The Late, 210
Balloons, Dangerous, 466
Barnes Urban District Council, 116, 178
Barling, Sir Gilbert, 281
Baschurch Surgical Home, 94
Bath Union Infirmary, Supp., Feb. 5
Baxter, Mr. Wynne, 24
Beatson, Sir George, 116
Bedford Hospital Fund, 425
Belfast University, 92
Belgian Practitioners, 373
Bermondsey: Guardians, 127, 591; Lying-in Hostel, 589
Biggar, Miss M. M., 568
Billericay Guardians, 555
Birkenhead Guardians, 425
Birmingham: Hospitals League, 25; District Nursing Institute, 85, 322; Hospital Saturday, 210; Chamber of Commerce, 281; Dudley Road Hospital, 547
Birth-Control, American Women and, 123, 431
Birth-Rate (*see* Infant Welfare)
Bishop Auckland Isolation Hospital, 412
Blackburn Hospital Fund, 331
Black-Coated Worker, The, 466
Blind, The: Committee on Causes of Blindness, 3; Registration of Charities, 3; The D'Albe Appliance, 498
Board of Education, Oct. 2, x, 195, 350
Boiler Explosion, 445, 466
Bonham-Carter, Mr. Henry, The Late, 577
Book World, The (*see* Institutional Library)
Books for Sick Men, 220
Bournemouth's Christmas-Box, 190
Bradford: Municipal Hospital, 23, 161, 380, 400, 478
Bread, 108
Brentford Guardians, 598
Brighton: Boxing Tournament, 378; Prince of Wales's Visit, 423
Bristol, A Pay Hospital for, 308; *The Medico-Chirurgical Journal*, 446; Babies' Home, 504
British Commercial Gas Association, 102
British Hospitals Association, 23, 93, 113, 144, 209, 247, 254, 336, 438, 468, 490, 512, 554, 597
British Medical Association, 128, 253, 281, 304, 327, 351, 482
British Women's Hospital Committee, 479, 596
Budapesth Hospital, In a, 362
Buncombe, Dr. W. B., 425
Burnett, Sir Napier, 5, 9, 53, 364

Camberwell Guardians, 110, 128, 456
Cambridge, The Position of Women at, 97
Canadian Army Nursing Service, 226
Cancer, 573
Cantlie, Sir James, 145
Cardiff: Mental Hospital, 124; Prince of Wales's Hospital, 188, 232; Health Committee, 321
Cardigan, New Hospital for, 444
Cardwell, Miss, 394
Carlogie House, Fire at, 189
Carrier Question, A, 165
Casual Help and Weekly Gifts, 209
Cave, Lord, 353 (*see also* Committee of Inquiry)
Cavell, Edith, 61, 851, 298; Homes, 369
Cenotaph, The, 161, 209
Census, The, 348, 411
Central Council for District Nursing, The, 149, 154, 526
Central Midwives Board, 392; for Scotland, 202, Nov. 27, x, 435
Chalmers, Dr. A. K., 191
Chapel-en-le-Frith Guardians, 298
Chaplains: Salaries, 4; In Asylums, 355
Chartered Society of Massage, 35, 370
Chelsea Nurses' Club, 348
Chepstow, A Hospital for, 308
Children's Rescue Fund, 151, 189
Child Welfare (*see* Infant Welfare)
China, Medical Education in, Feb. 19, vi, 493, 515
Chocolate Peril, A, 162
Church Missionary Society, 298
Christmas: An Opportunity, 251; Fellowship of, 279; Appeals, 275; Hospital Christmas, 288, 305; The Festival of Healing, 295; Gifts per Fire Engine, 332
Cinema, Children and the, 44; Hospital Funds, 70; Crime Films, 103; Propaganda Films, 338; The Film in Schools, 495
City of London Guardians, 128
Civic Education League, 430
Cleanliness by Law, 337
Clearing the Ground, 412
Cleaver, Mr. H. P., 444
Clinical Thermometers, 528
Clothes, Concerning, 3
Clothing and Health, 519
Coal Strike, The, 67, 81
Coles, Mr. R. J., 151, 187
**COLLEGE OF NURSING** 83, 125, 126, 149, 158, 175, 184, 198, 268, 271, 300, 368, 433, 567
Affiliated Training-Schools, 224
Elections, 391, Jan. 29, vi
General Meeting, 107, Oct. 30, vi, 151, 155, 204
Irish Board, Oct. 2, x, 42, 61, 397
Minimum Salaries, 367, 373, 456, 482
Nurses' Club, 62, Oct. 23, vi, 150, 225, 414, 456, 547
Studentships, 60, 61, Oct. 16, vi
Subscriptions, 106

COLLEGE OF NURSING—*continued.*
*The Bulletin*, 177, 502
The Register, 17, 225
CENTRES:
Ayr, 456
Belfast, 397
Birmingham, Oct. 2, x, 42, Oct. 16, vi Oct. 30, vi, Nov. 6, viii, 184, Nov. 29, x, Dec. 4, viii, 250, Jan. 29, vi, Feb. 5, vi, 456, 574
Brighton, Feb. 5, vi, 574
Bristol, 300
Cambridge, 397
Cardiff, Feb. 5, vi
Durham, Feb. 26, viii, March 12, iv, 600
Edinburgh, 42, Nov. 6, viii, 184, 274, Jan. 15, vi, 456, Feb. 26, viii, March 12, iv, 600,
Glasgow, Jan. 29, vi, Feb. 26, viii, March 12, iv, 600
Halifax, 346
Leeds, 350, Jan. 29, vi, 440, Feb. 26, viii, March 12, iv, 574
Leicester, 397
Liverpool, Jan. 29, vi, March 12, iv
London, Jan. 15, vi, 397, Jan. 29, vi, 434, Feb. 5, vi, Feb. 26, viii, March 5, vi, March 12, iv, 574
Manchester, 440, Feb. 26, viii
Norwich, Feb. 5, vi, 456, Feb. 26, viii, March 12, iv
Sheffield, 397, 440, March 5, vi, March 12, iv
Swansea, Oct. 16, vi, 397, 440
Torquay, Oct. 30, vi
Truro, 456, 600
COMMITTEE OF INQUIRY INTO HOSPITALS, 351, 353, 376, 390, 421, 423, 443, 507, 509, 575, 597, 599
Collet, Dr. G. B., The Late, 256
Colour-Blindness, 139
Coming of the Mob, The, 593
Continuation Schools, 474
Co-operative Buying, Supp., Oct. 16
Co-operation or Chaos, 327
Connaught, The Duke of, 356
County Areas, Medical Services in, 168
Coventry Saturday Fund, 163
Cowdray, Lord and Lady, 392, 414, 547
Crewe, Mrs., 226
Crime, The Prevention of, 539; Anonymity for Young Criminals, 590
Crisis, Two Chairmen on the, 186
Croydon: Infectious Hospital, 332; Saturday Fund, 527

Dale, Mr. Guy, Death of, 254
Dangerous Drugs Act Regulations, 51, 386, 425, 477, 482, 487, 510, 531, 563
Davidson, Miss F. M., 16
Dawson, Lord, 231
Daylight Saving Act, 94
Deane, Col. A., 68
Death and the Doctors, 76
Denmark, Hospitals in, 294
DENTAL: School Children, 173, 264; Army Dental Corps, 404; The Dentists Bill, 511, 542
Devonport Guardians, 107, 347, 509
Diamond, Sir W. H., 379
Diet and Hygiene Campaign, 540
Dill, Dr. Gordon (*see also* Provident Plans), 377, 466
Diphtheria, 112, 206, 447
Discharged Disabled Soldiers (*see also* Ministry of Pensions), 322
Discussion of a Policy, The, 328
DISPENSING (*see also* Drugs, and Dangerous Drugs Act): Hospital Officers' Association, 82; Pharmacy Examiners, 148; Should Medical Practitioners Dispense? 358; Caveats for Dispensers, 494
District Nurses, 62, 149, 523, 526
Dorset Hospital Association, 284
Dried Milk, 535, 581
Drugs (*see also* Dispensing), The Week's Market in, 6, 26, 48, 70, 94, 116, 138, 164, 190, 212, 234, 256, 284, 308, 332, 356, 380, 404, 446, 467, 489, 511
Drunkenness, The Decline of, 330
Duke of York, 462, 487
Durham: Domestic Science Centre, 462; County Health Committee, 525
Dust on the Underground, 22
Dysentery, 466

Eden, Dr. T. W., 284
Edinburgh: Medical Missionary Society, 211
**EDITOR'S LETTER-BOX, THE:**
Almoner Question, The, 592
Ambulance Facilities and Telephone Facilities, 477
Association of Certificated Blind Masseuses, The, 154
Best Form of Medical Service, The, 417, 437, 499
Birth-rate Factors, 372
Blind Masseurs, 179
Cambridge, The Position of Women at, 129, 153, 228
Coming of the Mob, The, 565
Dangerous Drugs Act, 477
Feeble-minded, Control of the, 522
Foes of Hospital Economy, 153, 203
Future of the Hospitals, The, 203
Gift of Expression, The, 19, 39, 63
Great Northern Central Hospital, 63
Health and Artistry, 109
Hours and State Control, 324
Housing the Nursing Staff, 272, 300, 324
"How Long Halt Ye?" 227, 245, 272, 300, 323, 349, 371
Income Tax on "Profits" of Charities, 153
Labour Ministry and Unemployment Insurance, The, 349
Living-out System, The, 272
London Regional Committee, The, 246
Medical Service for All, A, 299
Mental Treatment for Clinic, 245
"Monsters," 372
National Health Insurance Surpluses, 477
Nurses and Trade Unions, 458, 477
Nurses' Co-operation, The, 228
Old Comrades' Association, An, 179
Open Letter to Private Nurses, An, 299
Patients' Payments, 19, 109, 179
Private Hospitals and Trained Nurses, 543
Private Nurses and Hours of Employment Bill, 349
Private Nurses' Fees, 499
Psycho-Analysis, 371
Radiant Motherhood, 180, 246
Smoking by Nurses, 566, 592
Social Service in America, 566
Some Early Reports, 499
Spinal Anæsthesia, 372
Teacher or Nurse, 522
Unemployment Insurance, 592
Unmarried Mother and Her Child, The, 228
Voluntary Staff Conference, 543
Warwickshire's Record, 130
Edmonton Infirmary, 48
Education of Nurses, 347, 545; In Army Hospitals, 361; The Working-Class Boy, 364
Efficiency Exhibition, The, 443, 463, 471, 482
Egypt, Government Hospital for, 168
Eight-Hours Day, 59, 479
Emigration, The Physical Factor in, 389
Empire Hospital Fund, An, 233
Encephalitis Lethargica, 444
Enquiry, The Proposed Hospital, 160
Essex County Council, 190
Eugenics, The Dean of St. Paul's and, 206; In the School, 338
Everybody's Day (*see* Red Cross)
Everyday Medical Problems, 401, 405, 447
Expense of the Sick, The, 232
Experiments on Animals, The Value of, 71
Ex-Service Men's Carnival and Exhibition, 188
Ex-Service Welfare Society, The, 522

Fair and Dark, 448
Factories, 57, 62, 221
False Pretences, Obtaining Relief by, 430
Fasting, Suicide by, 91, 99
Fatigue, 453, 550
Federation of Medical and Allied Societies, 52, 327, 556
Fellowship of Medicine, 354
Fever Epidemic, The, 112
Fever Nurses: In Scotland, 176; Salaries, 393, 398
Finance of Hospitals, The Resilience of, 230
Finsen-Light Centres for London, 573
Fletcher, The late Mr. Alfred, 509
Fletcher, Miss Nora, 569
Flogging, The Futility of, 563
FOOD (*see* also Milk and Cookery): Contamination in Street Markets, 47; Dried Fruit and Vegetables, 50 (Supp., October 30); The Food Peril, 162; Hospital Bills, 321; Poisoning, 424
Football, Hospital Saturday, 511
Forrest, Col. J. V., 329
Forthcoming Events, 398, 420, 438, 477, 528, 560
Free Home for Nurses, 321
French War Emergency Fund, 38

Galsworthy, Mr. John, 33
Garrick Theatre Toy Distribution, 305
General Medical Council, 207
General Nursing Council, 15, 19, 37, 42, 85, 87, 126, 127, 181, 199, 269, 273, 347, 415, 434, 455, 459, 502, Feb. 26, vi; 567; Scotland, 250, 420, 434, 596; Ireland, 546, 548
General Practitioner, The, 90
Gibson, Dr. William, 234
**GIFTS AND BEQUESTS**, 40; Oct. 23, vi; Oct. 30, vi; 77, 205, 318, 330, 377, 395, 438, 498, 572, 599
Gifts in Kind, 576
Glasgow: Retired Nurses' Home, 456
Gonorrhœa (*see* Venereal)
Goode, Miss Florence, 37
Goole Isolation Hospital, 93
Grades of Nurses, 319
Greenock Nursing Association, 548
Gruesome Controversy, 430
Guild of Health, The, 547

Hæmoptysis, The Localisation of, 382
Hammersmith Maternity Committee, 457
Hampshire County Nursing Association, 525
Hampstead Maternity Home, 151
Hants County Nursing Association, 178, 225
Harley Street Associations, 237
Harper, Mr. W. H., 255
Harveian Society, The, 281, 351, 426
Harvest Festivals, 52
Hartismere Guardians, 322
Hatch, Sir Ernest, 353
Health and Economy, 486
Health by Knowledge, 565
Health Visitors, 16, 84, 200, 226, 504
Heart: Statistics of Disease, 7; The Healthy, 558
Hertfordshire County Council, 307
Higginbotham Sick Poor Nursing Association, 321
Highgate Hill Infirmary, 480
History of Hospitals as Depicted by Engraved Views, 141, 215, 261, 354, 359

Holden, Miss M. I., 206
Holiday Camp Schools, 219
Honiton and District Rural Nursing Association, 69
HONOURS:
Investiture by the King, 548
Medals for Red Cross Workers, 6, 455
New Year Honours, 329, 346, 354
Order of the British Empire, 127, 177
Royal Red Cross, 127, 151, 243
Hookworm, 165
Hop-Pickers, Among the, 396
Horsley, Sir Victor, Memorial to, 350
**HOSPITAL AND INSTITUTIONAL NEWS** 3, 23, 46, 67, 91, 113, 135, 161 187, 209, 231, 253, 281, 305, 329, 401, 423, 443, 465, 487, 509, 531, 553, 577
Hospitals and Insured Patients' Fees, 529
**HOSPITALS, INSTITUTIONS, ETC.** (Poor Law, Municipal, and Temporary Hospitals *see* under individual names):
METROPOLITAN:
Alexandra Hospital, 189, 226
Bethlem Royal Hospital, 69, 216, 344
British Hospital for Incurables, Nov. 6, viii
British Hospital for Women and Babies, 77, 92
Cancer Hospital, 282
Central London Ophthalmic Hospital, 534
Charing Cross Hospital, 37, 167, 479
Chelsea Hospital for Women, 344, 348, 373, 571
Church Missionary Society, 503
City of London Hospital for Diseases of the Chest, 301
City of London Maternity Hospital, 512
Dr. Barnardo's Homes, 331
East London Hospital for Children, 404
Elizabeth Garrett Anderson Hospital, 177
Evelina Hospital, 37
Foundling Hospital, 215
General Lying-in Hospital, 38, 48, 512
German Hospital, 467; Feb. 26, vi
Great Northern Central Hospital, 40, 62, 77, 115; Nov. 6, viii, 202; Nov. 27, x, 271, 344, 353, 373, 378, 380, 458, 550
Guy's Hospital, 40, 108, 254, 307, 311, 571
Hornsey Cottage Hospital, 524
Hospital for Consumption, Brompton, 234, 342
Hospital for Diseases of the Throat, Golden Square, 113
Hospital for Sick Children, 37
King's College Hospital, 38, 111, 115, 128, 136, 151, 187, 283, 297, 343, 359, 393, 507, 531, 535, 555
London Homœopathic Hospital, 115, 136, 198, 342
London Hospital, 116, 126, 133, 135, 230, 238, 329, 341, 395, 445, 527, 529, 573
London Jewish Hospital, 553
London Lock Hospital, 189
London School of Tropical Medicine, 161, 573
London Temperance Hospital, 92, 344, 598
Metropolitan Hospital, 180, 254, 282, 378
Middlesex Hospital, 36, 198, 201, 359, 471, 508, 512
Miller General Hospital, 48, 297, 307
Mount Vernon Hospital, 580
National Hospital for the Paralysed and Epileptic, 93, 131, 158, 348, 373, 445, 504, 573
Paddington Green Hospital, Nov. 6, viii
Poplar Hospital, 579
Prince of Wales's Hospital, 209
Queen Charlotte's Hospital, 404
Queen Mary's Hospital, 202
Queen's Hospital for Children, 62, 201, 503
Ranyard Mission. 550
Royal Free Hospital, 17, 37, 341, 403, 445, 504, 520
Royal General Dispensary, The, 174

HOSPITALS, ETC.—*continued.*
Royal Hospital for Diseases of the Chest, 353, 378, 445, 466; Feb. 19, vi
Royal Hospital and Home for Incurables, 6, 40, 93, 301, 398. 554
Royal London Ophthalmic Hospital, Nov. 6, viii, 258, 512
Royal National Orthopædic Hospital, 26, 46, 67, 136, 598
Royal Surgical Aid Society, Feb. 26, vi
Royal Waterloo Hospital, 457, 580
Royal Westminster Ophthalmic Hospital, 446
St. Bartholomew's Hospital, 40, 113, 133, 141, 311, 395, 402, 414, 423, 483, 501, 525, 554, 571
St. Columba's Hospital, 16
St. George's Hospital, 69
St. Mark's Hospital, Feb. 26, vi
St. Mary's Hospital, 301, 311, 423, 471, 571, 573
St. Mary's Hospital, Plaistow, 329, 395, 534
St. Thomas's Hospital, 107, 131, 132, 142, 188, 311, 531, 571, 579
Samaritan Free Hospital, 571
Seamen's Hospital Society (*see also* London School of Tropical Medicine), 359, 510
South London Hospital for Women, 70, 86, 311
University College Hospital, 68, 580
West-End Hospital for Nervous Diseases, 569
Western Ophthalmic Hospital, 237
West London Hospital, 524
Westminster Hospital, 226, 465
Willesden Hospital, 307, 332
PROVINCIAL, SCOTTISH, AND IRISH:
Aberavon Hospital, 395; Feb. 26, vi
Aberayron Cottage Hospital, 380
Abertillery and District Hospital, 88
Alderley Edge Cottage Hospital, 70
Alton Crippled Children's Hospital, 344
Aylesbury, Royal Bucks Hospital, 86, 511
Barnsley, Beckett Hospital, 256
Bath United Hospitals, 377
Batley and District Hospital, 467
Belfast: Ulster Volunteer Force Hospital, 62; Royal Victoria Hospital, 68, 93, 271, 329
Birmingham: Children's Hospital, 94, 138, 404, 450; General Hospital, 115, 152, 231, 423, 580; Highbury Orthopædic Hospital, 178; Queen's Hospital, 342, 578; Hospital for Nervous Diseases, 550; Ear and Throat Hospital, 579
Blackburn Royal Infirmary, 467
Bournemouth, Boscombe Hospital, 138
Bradford Royal Infirmary, 478, 570
Brighton, Royal Sussex County Hospital, 308, 350, 580
Bristol: Maternity Hospital, 164; Children's Hospital, 244; Royal Infirmary, 331; General Hospital, 533
Cambridge, Addenbrooke's Hospital, 254, 342, 524
Canterbury, Kent and Canterbury Hospital, 114
Cardiff: King Edward VII. Hospital, 6, 26, 114, 182, 190, 211, 311, 379, 403, 457, 550; Royal Hamadryad Seamen's Hospital, 532
Carlisle, Cumberland Infirmary, 394
Chesterfield Royal Hospital, 3, 210
Chichester, Royal West Sussex Hospital, 116, 582
Clacton and District Cottage Hospital, 511
Coalfield Cottage Hospital, 555
Coventry and Warwickshire Hospital, 322, 416
Croydon General Hospital, 178
Cumberland Infirmary, 1. 9, 249, 255, 553
Dartmouth Cottage Hospital, 288
Devonport, Royal Albert Hospital, 107, 446
Dewsbury General Infirmary, 402
Dolgelly Cottage Hospital, 70

HOSPITALS, ETC.—*continued.*
Doncaster Infirmary, 283
Dundee Dental Hospital, 306
Durham County Hospital, 488
Edinburgh Royal Infirmary, 69, 137, 283, 413
Elswick Hospital, 462
Evesham Hospital, 211
Exeter, Devon and Exeter Hospital, 138
Festiniog Memorial Hospital, 234
Folkestone, Royal Victoria Hospital, 344
Glasgow Royal Infirmary, 467, 507
Gloucester Royal Infirmary, 151
Gosport Hospital, 467
Halifax Royal Infirmary, 255
Halstead Cottage Hospital, 190, 233
High Wycombe, Beaconsfield Memorial Hospital, 256
Holmesdale Cottage Hospital, 25, 370
Hove Hospital, 37
Ipswich, East Sussex Hospital, 92, 94, 109, 231
Keighley, Victoria Hospital, 344
Kilmarnock Infirmary, 467
Lancaster Royal Infirmary, 528
Leamington, Warneford Hospital, 241, 373, 488
Leeds General Infirmary, 256, 354, 415, 419, 433
Leicester Royal Infirmary, 25, 169, 194, 255, 334, 416, 571
Liverpool: Royal Infirmary, 187, 580; Royal Southern Hospital, 298, 393, 580; Dental Hospital, 488
Livingstone College, Dartford, 559
Llanelly Hospital, 462
Longton Cottage Hospital, 189, 233
Maidstone: West Kent General Hospital, 182, 554; Kent County Ophthalmic Hospital, 489, 579
Manchester: Ancoats Hospital, 137, 446; Royal Infirmary, 162, 211, 445, 507; Children's Hospital, Pendlebury, 397; Lock Hospital, 380
Mansfield and District Hospital, 350
Middlesbrough, North Ormesby Hospital, 233
Mountain Ash Hospital, 533
Newcastle-on-Tyne, Royal Infirmary, 70, 107, 119, 509
Newport, Royal Gwent Hospital, 403, 554
Newton Abbot Hospital, 284
Northampton, General Hospital, 188, 356, 468
Norwich, Norfolk and Norwich Hospital, 69, 211, 490
Nottingham, General Hospital, 26
Oxford, Radcliffe Infirmary, 3, 162, 183, 283, 393, 568
Peterborough Infirmary, 25
Pontypridd Cottage Hospital, 178, 467
Portsmouth, Royal Hospital, 271, 331, 492
Ramsgate General Hospital, 189, 370
Redruth Hospital, 210
Ripon Cottage Hospital, 489
Rochester, St. Bartholomew's Hospital, 138
Salford, Royal Hospital, 158
Scorton, Hospital of St. John, 533
Sheffield, Royal Infirmary, 61, 128
Stamford, General Infirmary, 481
Stoke-on-Trent, North Staffordshire Infirmary, 244, 403
Stourbridge and Halesowen: Hospital, 18; Cottage Hospital, 25
Sunderland, Royal Infirmary, 38, 445, 507
Swansea Hospital, 380, 578
Taunton and Somerset Hospital, 332
Trowbridge Hospital, 297
Truro, Royal Cornwall Infirmary, 402
Tunbridge Wells, General Hospital, 330, 425
Wakefield, Clayton Hospital, 178, 445, 548
Wallasey, Cottage Hospital, 307
Walsall, General Hospital, 532
Welshpool Hospital, 457

Hospitals, Etc.—*continued.*
West Hartlepool, Cameron Hospital, 579
Weymouth, Royal Hospital, 40
Wigan, Royal Albert Infirmary, Feb. 12, viii
Whitehaven Infirmary, 403
Winchester, Royal Hants County Hospital, 256, 489
Windsor, King Edward VII. Hospital, 403
Wolverhampton, General Hospital, 255, 344, 356; Feb. 26, vi
Worcester General Infirmary, 354
York County Hospital, 489, 598
Colonial, U.S.A., etc.
Assam, Shillong Hospital, 96
Christchurch (New Zealand) Hospital, 456
Grahamstown, Albany General Hospital, 553
Johannesburg, General Hospital, 384
Melbourne, Alfred Hospital, 118
Montreal: General Hospital, Western Hospital, 555
New York, Fifth Avenue Hospital, 114
Peking Union Medical College, 515
St. Helena Hospital, 384
Shantung University Hospital, 516
Sydney, Royal North Shore Hospital, 30
Transvaal, Boksburg Hospital, 226
Hospital Officers' Association (*see* Incorporated)
Hospital Saturday Funds, 26, 46, 92, 283, 328, 395, 424, 462, 487, 577
Hospitals in Literature, 562
Hospital Sunday Fund, 282
Hostels for Professional Women Supp. March 19
Hours of Employment Bill, 87, 199, 368
Housekeepers' Store Cupboard, Supp. Oct. 16
Housing: First Eastern General Hospital, 25; Housing and Health, 387; Child Welfare, 587
Howard, Mr. R. J. B., The Late, 356
How Goes the Fight? 507
How Not to Eat, 212
How Old is a Man at Forty? 335
Hudson, Sir Robert, 306
Hull Corporation and Crèches, 176
Hygiene, Army Schools, 432

Iceland, Hospital Progress in, 532
Identification, Novel Means of, 308
Ilford Maternity Home, 395
Illegitimacy, National Council, 80
Ince, R. B., 110
Incorporated Association of Hospital Officers, 64, 82, 301
Incorporated Society of Trained Masseuses (*see* Chartered Society of Massage)
Incrustations in Soil-Pipes, 194
India: Hospital Problems in, 14, 98, 362; Nursing, 18; Conditions of Childbirth, 582
Industrial Health, 147
Industry: Women in, 172; The Human Factor in, 541
Infantile Acidosis, 235
Infant Mortality in Europe, 121, 431
INFANT WELFARE AND MATERNITY: (*see also* Midwives, and the Public Well-Being); Ill-nourished Children, 4; Llanwit Vadre Centre, 6; Birth-Rate, 24, 32, 54; Psychology of Children, 55; Baby Week, Oct. 16, vi; Breast Feeding, 121; Dr. Addison and Cottage Nurses, 175; The Future of the Crèche, 176; Sir James Cantlie, 183; Administration of Act, 206; Nutrition, 291; Southwark, 297; Nursery Schools, 315; Post-Graduate Course, 373; Mortality, 431, 573; The Right of the Child, 473; Poverty and the Baby, 496; Inspection of Children in Hampshire, 527; Essex, 573; Housing and Child Welfare, 587; List of Centres, 598
Infectious Diseases, Fever Increase in London, 24
Influenza, 377, 381, 424, 443, 453
Inquiry Officer, The Work of an, Supp. March 5
Insane (*see* Mental)
Institute of Hygiene, 558
INSTITUTIONAL FACTS AND FIGURES (*see* The Institutional Worker Supplement to "The Hospital")
INSTITUTIONAL LIBRARY:
Ballantyne: A Pocket-Book of Ophthalmology, 314
Bardswell: Handbook for Tuberculosis Workers, 313
Blackham: Care of Children, 58, 505
Black's Simple Cookery, 505
Bradford: A Hospital Letter-Writer in France, 18
Brainard: Organisation of Public Health Nursing, 218
Burdett's Hospitals and Charities, 399
Carter: Elements of Medicine, 521
Clayton: Medical Gymnastics in Medicine and Surgery, 41
Cooke: A Nurse's Handbook of Obstetrics, 505
Cope: The Surgical Aspects of Dysentery, 586
Cowan: Bandages and Bandaging for Nurses, 570
Davies: A Son of Life, 104
Davis: Hypnotism and Treatment by Suggestion, 500
Dewberry: The Prevention and Destruction of Rats, 314
Dock: A Short History of Nursing, 505
Donnell: Letters of an Australian Army Sister, 374
Dulberg: Sterile Marriages, 120
Dysentery, Notes on (Ministry of Pensions) 314
Farr: Internal Medicine for Women, 374
Fortescue-Brickdale: A Text-book of Pharmacology for Nurses, 544
Gallichan: The Critical Age of Women, 170
Godlee: Six Papers by Lord Lister, 584
Goodall-Copestake: The Theory and Practice of Massage, 505
Groves: A Synopsis of Surgery, 314; Surgical Operations, 374
Gullan: Theory and Practice of Nursing, 201, 268
Halliburton: Handbook of Physiology, 500
Hospital Surgeon, Memories of an, 521
Howard: Surgical Nursing, 218
Hunter: Mechanical Dentistry, 170
Hutchison and Rainy: Clinical Methods, 218
Ibbotson: Atlas of Cutaneous Nerves, 158, 521
Infant Weight Chart, 521
Ker: Infectious Diseases, 120
Kerr: Guide to Industrial Welfare Work, 314
Kidd: Common Infection of the Kidneys, 10
Knox: General Practice and X-Rays, 521
Kynaston: Adenoids and Enlarged Tonsils, 170
Lane-Claypon: The Child-Welfare Movement, 374
Lumb: Gonococcal Infection in the Male, 10
Lord: The Story of Horton War Hospital, 561
Mann: Text-Book of Tracheo-Bronchoscopy, 586
Manson: Tropical Diseases, 559
Manual of Public Health, 586
Marchant: The Control of Parenthood, 79
Institutional Library—*continued.*
Martin: Genito-Urinary Surgery, 500
Marx: Woman, 586
Maublanc: The Medical Examination of Airmen, 521, March 12, iv
Meachen: Tuberculosis, 120
Medical Directory, 362
Milnes, Nora: Child Welfare, 587
Moore: This Concerns You, 586
"Nursing Mirror" Pocket Encyclopædia, 374
Osler: The Principles and Practice of Medicine, 313
Parker: Early History of Surgery in Great Britain, 170
Piersol: Normal Histology, 521
Reid: Prevention of Venereal Disease, 218
Rideal: Approved Technique of the Rideal-Walker Test, 585
Robertson: A Manual of Medical Jurisdiction, 586
Robinson: Cunningham's Manual of Practical Anatomy, 10
Rowes and Orr: Functional Mental Illnesses, 313
Samey: Personal Hygiene, 170
Scott: Why Do We Die? 544
Stenhouse: Health Reader for Girls, 41
Stopes: Radiant Motherhood, 120; Truth about Venereal Diseases, 585
Sutton: Selected Lectures and Essays, 314
Tayler: A Scottish Nurse at Work, 41
Thomas: The Education Act, 1918, 170
Tidy: A Synopsis of Medicine, 500
Timberg: Home Exercises for Spinal Curvatures, 313
Tubby: A Consulting Surgeon in the East, 293
Tunstall: First Aid to the Injured and Sick, 544
Vivian: Lectures to Nurses in Training, 178
Von Reuss: The Diseases of the New-born, 585
Walker: Intestinal Disinfection, 120, 585
Ward: Rhoda Drake, 41
Watson: Handbook for Nurses, Jan. 29 viii, 570
Watson: A Complete Handbook of Midwifery, 544
Webb: Electro-Therapy, 314
Whittaker: Catechism Series: Surgical Anatomy, 500
Willis: Wedded Love or Married Misery, 120
Woodhead: Industrial Colonies for the Consumptive, 344
Woodwark: Manual of Medicine, 218
Insurance Scheme for Hospitals, 212
International Journal, A New, 162
International Medical Degree, An, 43
International Red Cross, 55
Invalid Children's Aid Association, Nov. 6, viii, 172, 197
Invalid Kitchens of London, 201
Irish Public Health Council, 46
Islington: Borough Council, 307; Infirmary, 415, 435
Isolation Hospitals, 191

Jenner, Lady, 54
Johncock, Sister Edith, 480
Johnson, Mr. T. F., The Late, 591
Jones, Miss B. L., 435
Journalism, New, How to Use the, 441

KING, THE: "Our Day," 46; Opens Parliament, 465

King Edward's Hospital Fund for London, 65, 75, 254, 265, 289, 361, 421, 439, 462, 558
King's College for Women, 38
Knaresborough Guardians, 547

Labrador, St. Anthony's Children's Home, 532
Lady Minto's Indian Nursing Association, 369
Lambeth: Guardians, 52; Borough Council, 84, 85, 554; Appeal to the Home Secretary, 531
Laundry Exhibition, 202
Lay Newspapers and Medical News, 306
League of Mercy, 188, 282, 294, 325, 576
League of Nations, International Health, 171, 177
League of Remembrance, 201, 220
"Leavings" for the Hospitals, 47
Leeds: Flag Days in, 47, 91; University Diplomas in Nursing, 433; Trained Nurses' Institute, 481
Leicester: Hospital Accommodation, 25; Hospital Saturday, 443
Leprosy: Indian Lepers Act, 5; The Problem in India, 383
Lewes Guardians, 322
Leyton District Council, 462
Leytonstone Hospital Society, 462
Lifeboat Ambulance, A, 162
Lincoln: Board of Guardians, 17, 86; Nursing Association, 86
Liverpool: Council of Voluntary Aid, 533
Living-in, The System of, 223
Lloyd, Mrs. E., 177
Local Government Board (*see* Ministry of Health)
London County Council, 105, 217, 170, 315, 350, 379, 481, 584
London Medical Exhibition, 29
Loughborough Nursing Association, 108
Lyle, Mr. C. E. Leo, 569

MacCallum *v.* Burdett and Others, 206
Macfie, Dr. R. C., 99
McIlroy, Dr. Louise, 503, 520
Mackintosh, Col. D. J., 26
Madness (*see* Mental)
Malaria, 573
Male Nurse, Charge against a, 394, 596
Malmesbury, The Earl of, 395
Manchester: Three Thousand More Beds, 163; Charities Fund, 307; Communal Nursing Scheme, 320; Insurance Companies, 355; Hostility to Hospitals, 379; Hospital Co-operation, 401; Infirmary Paying Patients, 509
Marriage Law Reform, 552
Marsden, William, 305
Marylebone: Borough Council, 162; Infirmary, 394
Massage and Medical Gymnastics, 345
Maternity: (*see also* Infant Welfare and Midwives); Work at Woolwich, 4; Working Women, 101; The Lying-in Period, 212; Wards for Sick Poor, 355; Municipal Contributions, 404; Fees, 424
**MATRONS' AND SISTERS' DEPARTMENT**, 15, 35, 59, 83, 105, 125, 149, 175, 199, 223, 243, 269, 295, 319, 345, 367, 391, 413, 433, 455, 479, 501, 523, 545, 567, 593
Measles, 379
Medals (*see* Honours)
Medical Appointments at Hospitals, 578
Medical Future, The, 207
Medical Guild in Scotland, 401
Medical Officers of Schools Association, 274
Medical Research Council, 333, 378
Medical Scholarships, 403
Medical Schools: Reopening, 30; Government Grants, 274; Magazines, 571
Medical Service for All, A, 230
Medical Society of London, The, 66
Medical Standpoint, The, 304
Medicine: Many Years Ago, 259; The Collective Voice of, 556
Medico-Political Union, 327
Medico-Psychological Association, 595
Meldrum Forced-draught Furnace, The, 543
Memorials: To War Nurses, 297; To All Nurses, 369
MENTAL: Treatment for Crime, 167; Incipient Diseases, 208; Home for Afflicted Officers, 233; Report on Brighton Hospital, 298; An Overdue Reform in, 330; Nurse Training, 501
Mercier, Dr. Charles, The Late, 531
Mesopotamia, Nursing in, 435
Metropolitan Asylums Board, 17, 435, 538
Metropolitan Water Board 113
Middle-aged Man, The, 388
Middle Classes Union, The, 578
Middlesex County Council, 78
Midwives: Durham County Inspector, 178; Midwives (Scotland) Act, 436, 504
Midwives' Institution, 370, 570
Milk, 13, 81, 95, 232, 472, Feb. 19, vi, 535, 581
MINISTRY OF HEALTH: Miscellaneous Provisions Bill, 25, 52, 91, 111, 133, 135, 161, 187, 207, 209, 252, 253, 280, 486; Sir G. Newman, 73; Medical Staff, 114; First Annual Report, 117; Future of Hospitals, 217; Consultative Council, 231; Maternity and Child-Welfare Centres, 253; Neath Hospital, 298; Report on Islington Infirmary, 415; Influenza Report, 424; Food Poisoning, 424; General Nursing Council, 455; Sir Kingsley Wood, 475; Committee on Vaccines, 487; Infirmaries and Paying Patients, New Bills, 550; Welsh Consultative Council, 555; List of Welfare Centres, 598; Medical Staff, 599
Ministry of Labour, 244
Ministry of Munitions, 466
Ministry of Pensions, 595
Misunderstood, 578
Money, Sir Leo, 92
Monk, Miss Beatrice, 296
Morality and Marriage, 196
Morley, Mr. Arthur S., 136
Morphine Poisoning, 406
Morris, Mr. E. W., 283
Mortuary, The Hospital, Supp. Oct. 30,
Motherhood (*see* Infant Welfare)
Motor Show, The, 143
Moynihan, Sir Berkeley, 419
M.P.s on the Hospital Question, 47
Multitude of Words, The, 174
Municipal Convalescent Home, A, 307
Municipal Representation on Hospital Committees, 395
Mysterious Visitor, The, 46, 91

National Association for the Prevention of Infant Mortality
National Association for the Prevention of Tuberculosis (*see also* Tuberculosis), 3, 429
National Council of Women, 88
NATIONAL HEALTH ASSURANCE: Divisional Medical Officers, 206; County of London Insurance Committee, 226; Some Curious Points, 234; Passing of Local Committees, 305; Panel Medicines, Jan. 1, iv; Doctor and Patient, 339; "Panel Prying," 364; Secrets of the Panel, 409; Sanatorium Benefit in Scotland, 438; Accumulated Funds, 464, 529, 575, 578; Record Cards, 454, 487, 518, 599
National Relief Fund, 46, 206, 209, 230, 329, 377, 532, 553, 572
National Society for the Prevention of Infant Mortality, 317, 347
National "Unfits," 263
Nation's Fund for Nurses, 202, 244, 456, 479, 506, 569, 573, 596
Navy Surgeons' Fees, 528
Neal, Dr. J. Breward, 379
Newcastle: City Council, 128; University, 423
Newman, Sir George, 73
Newport, Fire at Alteryn Fever Hospital, 402
New Sources of Revenue, 136
Newton Abbot Guardians, 284
New Year, A Policy for the, 303
New Zealand: Resettlement, 200; Massage, 548
Noise and Health, 240
Northampton Saturday Fund, 468
Norwich Saturday and Sunday Funds, 577
Northumberland County Nursing Association, 86
Nosocomia Freemasons' Lodge, Jan. 15, vi
Nurse and Her Advocates, The, 340
Nurse, How Many Beds to Train a, 270
Nursery Schools, The Importance of, 495
Nurses' Co-operation, The, 83, 223, 568
Nurse's Library, A, Supp., Dec. 25, Jan. 22
Nurses' Missionary League, 17, 547
Nurses' Needlework Guild, The, 222, 268
Nurses' Resettlement Department, 60
Nursing Centre for the Empire, 392
Nursing Hours and Nursing Institutions, 318
Nursing Services, The Economic Development of the, 249
Nursing the Baby, 197

**OBITUARY**: Miss F. M. Davidson, 16; Mr. Wynne Baxter, 24; Miss Florence Goode, 37; Mr. A. C. Wickins, 81; Miss H. H. Rucke-Keene, 202; Mr. William Baker, 210; Mr. Guy Dale, 254; Mr. J. S. Wood, 282; Countess Roberts, 296; Dr. George Peacocke, 321; R. J. B. Howard, 356; Dr. J. Breward Neal, 379; Miss B. I. Jones, 435; Sister Edith Johncock, 480; Mdlle. Thulliez, 503; Mr. A. Fletcher, 509; Miss Lucy Robinson, 546; Lady Henry Somerset, 568; Mr. H. Bonham-Carter, 577; Mr. T. Fielding Johnson, 591
O'Connor, Dr. Brodie, 62
Old Age and Death, 195
Old Order Changeth, The, 242
Open-air Schools, 195
Opium, Morphine, and Cocaine, Exports of, 294
Organo-Therapy, The Value of, 285
Orthopædic Surgery, The Future of, 258
"Our Day," First Fruits of, 306
Overseas, A Lesson from, Supp., Dec. 11, 1920
Oxygen, Medical Uses of, 491

**PARLIAMENT AND THE PROFESSIONS**, 206, 294; Jan. 1, iv, 528, 550, 573
Parliament, New Session, 465, 598
**PASSING EVENTS AND TOPICS**, 40; Nov. 6, viii, 153, 198, 350, 373, 395, 462; Feb. 9, vi; Feb. 26, vi, 527, 550, 573, 598
Patients' Payments, 89, 210, 451; Doctor's Fees 442
Patients' Waiting-Lists, Supp., Nov. 13, 27
Pay of the Nurse, The, 523, 545, 593
Peacocke, Dr. George, The late, 321
Penal Reform League, The, 293
People's League of Health, The, 306, 454, 589
Personalia, 184, 238; Jan. 15, vi, 398; Feb. 12, viii, Supp., March 5, 2, 543, 572, 600
Physical Training in Continuation Schools, 317
Physics, Lectures on, 395

Pitt, G. H., 553
Plague and the Rat, 146
Plymouth Ambulance Station, 298
Poisons and Pharmacy Act, 340
POOR LAW: Future of Infirmaries, 52, 253, 281, 426; Hospitals under Voluntary Committee, 53; Extravagance, 108; New Institutional Dietary, 163; Workhouse Waste, 221; New Era for Nurses, 243; Relief, 350; Paying Patients in Infirmaries, 356, 379, 386; Reform, 452; Expenditure under, 550
Portsmouth Guardians, 348, 546, 569
Post-Graduate Medical Education, 354, 428
Preston, Mr. H. J., 378
Preventive Medicine, An Instrument for, 73
Prescription Writing, 310
Prince of Wales, 45, 46, 209, 226, 377, 423, 465
Princess Alice's Home, Slough, 152
Princess Mary, 37
Prisons Nursing Service, 502
Private Hospitals, 432, 506
Private Nursing Fees, 413, 545
Probationers: Age, 35; Advertising, 36; State Control, 39; Scarcity, 38; Test for Candidates, 59; A Higher Curriculum, 523
Professional Secrecy, 353
Professional Spirit, The Dawn of the, 345
Professional Women, Hotels and Clubs for, Supp., Jan. 8
Propaganda, Concerning, 14, 34
Proprietary Medicines Bill, 550
Provident Plans for Paying Patients, 377, 385, 401; Jan. 29, viii, 466, 511
Psycho-Analysis, 162, 257
Psychological Research, 401
Public Education, 485
Public Health: Should the Services be Scrapped? 409; In the Near East, 466
Public or Private Schools, 517
Public Pharmacists' Association, 130, 236, 454
**PUBLIC WELL-BEING, THE**: 11, 31, 55, 79, 101, 121, 145, 171, 195, 219, 239, 263, 291, 315, 337, 363, 387, 409, 429, 451, 473, 495, 517, 539, 563, 587
Puerperal Sepsis, 27

QUEEN, THE: "Our Day," 46; St. Bartholomew's, 414, 483, 501
Queen Alexandra's Imperial Military Nursing Service, 127; Dec. 4, viii, 503, 595
Queen Mary's Hospital, Roehampton, 47
Queen Mary's Hostel,
Queen Mary's Needlework Guild, 151, 177
Queen Victoria's Jubilee Institute for Nurses, 321, 456; Scottish Branch, 320
Quennell, Mr. Sidney, 465

Recent Official Publications, 39, 590
RED CROSS: (*see also* V.A.D.), Red Cross Week and Our Day, 5, 17, 21, 23, 46, 47, 67, 91, 113; Scheme of Distribution, 23, 33; Nurse Fined, 38; Ambulances and the Strike, 68; Conference at Cardiff, 96; The Cenotaph, 209; League of Mercy, 295; Library, 394, 573; Priory for Wales, 446; Sale of Government Stores, 466; The Medal, 455; Financial Report, 487; Edinburgh, 522; League of Red Cross Societies, 533; Armistice Day, 550
Regimental Debts Act, 348
Registrar-General's Report, The, 476
Renal Cases, The Prognosis of, 405
Research, Medical, 161, 192, 514
Rest and Recreation, 101
Risbridge Guardians, 504
Roberts, Countess, Death of, 286, 319
Robinson, Miss Lucy, The Late, 546
**ROUND THE HOSPITALS**, 16, 37, 61, 85, 107, 127, 151, 177, 201, 225, 243, 271, 296, 320, 346, 369, 393, 414, 434, 455, 480, 502, 524, 546, 568, 594
Royal Army Medical Corps, 93
Royal Army Service Corps Memorial Fund, 283
Royal College of Surgeons, England, 350
Royal Institute of Public Health, 223
Royal Medical Benevolent Fund Guild, The, 144, 254, 330
Royal Naval Memorial Fund, 177
Royal Sanitary Institute, The, 347, 395
Royal Society for the Protection of Birds, 226
Royal Society of Medicine, 161
R.S.O. *versus* Visiting Staff, 6
Rucker Keene, Miss H. H., The Late, 202
Russia, American Red Cross Relief, 18
Rutherford, Mr. Herbert F., 378
Ryan, Mr. Thomas, 424

Safety First Campaign, 12
St. Pancras Borough Council, 462
St. Barnabas Nurses' Guild, 394
Salford Guardians, 481
Salvarsan Substitute, Jan. 1, iv.
Sanitation, Problems of, 423
Scarlet Fever, 112, 206
School Children: London Hospitals, 100; The Best Discipline, 147; Health of, 173; Physical Tests, 429
School Teachers, The Physique of, 316
Scotland: Marriage Figures, 197; Voluntary Hospital Conference, 528
Scottish Board of Health, The, 543, 577
Scottish Matrons' Association, 152
Scottish Women's Hospital, 150
Secret Remedies of the Past, 74
Sera, The Standardisation of, 530
Service de Santé Militaire, 306
Severn Electricity Scheme, 240
Sheffield Sanatorium, 70; Joint Hospital Board, 425
Shepherd, Mr. A. F., 424
Shore, Miss F. N., 85
Shrewsbury Workhouse Infirmary, 395
Sickness and the New Poor, 220
Sites of Hospitals, Their Values, 162
Situations in London, 210
Sleaford Guardians, 18
Sleeping-Sickness, 389, 510
Sleep, 11
Small Holdings and Hospitals, 390
Smallpox, 137, 164, 294, 534
Smoking by Nurses, 549, 566
Social Notes, 183
Society of Radiographers, The, 436
Somerset, Lady Henry, 568
South Africa: News from, 384; Trained Nurses' Association, 394; Public Health Act, 481
South-East London Midwives' Association, 190
South Indian Nursing Association, 394
South London Nursing Association, 370
Southport, New Maternity Hospital, 457
South Wales Nursing Association, 415
Southwark: Guardians, 92, 108, 271, 525; New Hospital, 137; Borough Council, 297, 380
Spain, Foreign Degrees in, 198
Sphere-Gap Voltimeter, Supp. Dec. 11, 1920
Spinal Anæsthesia, 302
Spirit Duty, 40
Stanley, Sir Arthur, 5, 61
Star and Garter Hospital, 444
Starvation, 99
State Medical Service, A, 375
State Nursing Service, A, 82, 104, 124, 125
State Registration (*see also* General Nursing Council and College of Nursing), 15, 58
Stoke Board of Guardians, 308
Stoke Newington Day Nurseries, 598
Stopes, Dr. Marie, 123
Street Accidents and Poor-law Infirmaries, 163
Students' Aid to Hospitals, The, 445, 465
Sub-Acute Nephritis, Surgical Treatment of, 214
Sugar, 428
Suggestion as an Aid in Nursing, 110
Suicide of a Nurse, 202
Sunday Games, 497, 564
Surgical Education in the Sixteenth Century, 537
Surgical Failures, A Factor in, 357
Sussex Provident Scheme (*see* Provident)
Sutherland, Mr. James, 436
Swansea: Hospital Board, 69; Infirmary, 127; Health Committee, 321; Queen's Nursing Association, 415; Maternity Home, 598
Swedish Red Cross, 86
Swollen Glands, 353
Syphilis (*see* Venereal Diseases)

Team Work Among Nurses, 105
Technical Publishing, A Century of, 286
Telephones, 378, 528, 554
Territorial Force Nursing Service, 269
Three Towns Nursing Association, 86
Thulliez, Mdlle., 503, 525
"Tin-foil" Beds, 116
Tobacco Smokers, A Shock to, 122
Tonsils and Adenoids, 330
Trade Subscriptions, 130
Tredegar Medical Aid Society, 58
Tropical Diseases in the Navy, 492
Truro Nursing Association, 574
**TUBERCULOSIS**: (*see also* National Association); Liverpool Sanatoria, 4; Diagnostic Tests, 23; Mansions as Sanatoria, 26; Complement Fixation, 28; Sanatorium Gardeners, 48; Liverpool Conference, 51; Wider Difficulties of Treatment, 57, 388; Jugo-Slavia, 72; East Lancashire Service, 80; Heresies, 98; Conference, 107; Nottingham Sanatorium, 115; Topics, 119; Welsh National Memorial, 137; South Shields Sanatorium, 148; Lads' Sanatorium at Hale, 164; East Ham Sanatorium, 164; Welsh Chair, 189; The Anti-Tuberculosis Campaign, 193; Joint Disease in Children, 213; Sir Napier Barnett on Early Cases, 225; The Tuberculosis Society, 232; The Fight Against in London, 239; Notes, 260, 408; Stigma of the Dispensary, 332; Surgical Sanatorium, 355; Discharged Men, 366; New Sanatoria, 412, 461; Recent Work, 427; Registrar-General's Report, 444; Dispensary Survey, 461; Forthcoming Conference, 462; Anti-Tubercle Serum, 465; Lectures on, Feb. 19, vi; Winsley Sanatorium, 470; Spahlinger Cure, 492, 528; Smallpox or Tuberculosis, 534; International Conference, 549; Effect of War, 550; Age and Locality, 557; Dutch Anti-Tuberculosis Work, 583
Turbine Furnace, The, 287
Typhus, 55; in Poland, 525
Typist, The Hospital, Supp. Oct. 9

Uncertified Deaths, Jan. 1, iv
Unchampioned Nurse, The, 222
Underground Cafés, Health in, 212
Unemployment: Effect on Dispensaries, 577; Health Dangers in, 589
Unemployment Insurance, 106, 158, 205, Dec. 4 viii, 297, 367, 462, 594
Unfit, Multiplication of the, 588
United Services Fund, 480, 504
United States: Hospital System, 284; Medical Education, 403; The Social Worker, Feb. 26 viii, 570

University College, 365
Unknown Warrior, The, 135, 161
Unnecessary Noise, 134
Utility Bed, The, 436

Vaccination, 166
Vaccines, etc., Standardisation Act, 510
V.A.D.: (*see also* Red Cross); Red Cross Peace Work, 5, 46; Army Employment, 127
VENEREAL DISEASES: (*see also* National Council); Ministry of Health Report, 12; Problems, 31; Treatment of Syphilis, 49; Home for Children, 151, 189; Criminal Law Amendment Bills, 231; Liverpool, 316; An Example of False Morality, 451; Committee Report, 408, 508, 519; Treatment Centres, 573; Information for Nurses, 598; In the Army, 599
Verity, Mr. George, 167
Victory Ball, The, 244

Walker, Dr. Cranston, 377
Walker, Dr. Norman, 577
Walthamstow Linen League, 108
Waste, Checks on, Supp. Feb. 5, 19
Water, Cost of, 554
Watt, Miss A. J., 393
Weekly Contributions Extended in London, 159, 185
Welfare Work Among Boys, 517
Wellcome Historical Museum, 478, 598
Wellhouse Hospital, 578
Welsh National Memorial Association, 233
Welsh Hospital, Netley, 531
Western Fever Hospital, 350
Whipps Cross Hospital, 348
Whisky, The Prophylactic Value of, 294
White, Sir W. H., 66
Wickins, Mr. A. C., 91
Willesden: Hospital Accommodation, 34; Urban District Council, 548
Wills of Medical Men, 40, Oct. 23 vi, 324, 592
Witham Nurses' Association, 152
Women: and Public Health, 13; in Medicine, 24, 32, 68, 91, 132; and a New World, 291; in Industry, 520
Women Jurors, 410
Women Police, 525
Women's Imperial Health Association, 74
Wood, Mr. J. S., Death of, 282
Wood, Sir Kingsley, 475
Words and Deeds, 352
Workers' Leisure, 541
Workhouse Nursing Association, 595
Workmen's Compensation, 536
Wright, Sir Almroth, 161
Wright, the Rev. C. E. L., 233

X-Rays, 40

Year-Book of Charity, The, 399
York District Nursing Fund, 128
Yorkshire Voluntary Charities Association, 23

Zenana Bible Mission, Supp. Feb. 2

Printed by SPOTTISWOODE, BALLANTYNE & CO. LTD.

London, Colchester & Eton, England

### The College of Nursing

### Matrons' and Sisters' Appointments.

East Suffolk Hospital, Ipswich.—Miss Annie Taylor has been appointed theatre sister. She was trained at Leeds General Infirmary.

Infant Hospital, Dundee.—Miss Helen Edward has been appointed matron. She was trained at Dundee Royal Infirmary, and holds the certificate of the Central Midwives Board. She has also been assistant matron of King's Cross Hospital, Dundee, sister at Perth Royal Infirmary, at the Children's Hospital, Ashton-under-Lyne, and night sister at King's Cross Hospital, Dundee.

North Staffordshire Infirmary, Stoke-on-Trent.—Miss H. Griffith has been appointed night superintendent. She was trained at Chester Royal Infirmary, and has since been sister at Cambridge Military Hospital, temporary night sister and sister of the surgical ward at the David Lewis Northern Hospital, Liverpool.

Graymount Tuberculosis Hospital for Children.—Miss Violet Jolly has been appointed matron. She was trained at the Royal Victoria Hospital, Belfast, and at Lord Mayor Treloar Cripples' Hospital, Alton, in housekeeping. She has since been staff nurse at the Royal Victoria Hospital, Belfast, and staff nurse in Queen Alexandra's Imperial Military Nursing Service.

Cottage Hospital, Dolgelly.—Miss Margaret P. Williams has been appointed matron. She was trained at Withington Hospital, where she was later sister. She has also been sister at the Western Fever Hospital, and deputy-matron of Keighley Hospital. She has also done military work.

Isolation Hospital, East Ham.—Miss Annie Forrester Hindle has been appointed assistant matron. She was trained at the North Staffordshire Infirmary, and has since been night sister and acting assistant matron of Charing Cross Hospital and assistant matron of Cumberland Infirmary, Carlisle.

Borough Hospital, Burton-on-Trent.—Miss Kate Boyes has been appointed matron. She was trained at Liverpool City Hospital and Wolverhampton General Hospital. She has since been charge nurse at Boldon Fever Hospital, Sunderland, sister at Bolton Fever Hospital, night sister at Ipswich Borough Isolation Hospital, matron of Garnfield Isolation Hospital, Stone, and of Keynsham Isolation Hospital, and has done private and district nursing. Miss Boyes is a member of the College of Nursing.

Royal Infirmary, Preston.—Miss Mary Strachan has been appointed sister. She was trained at Dundee Royal Infirmary, where she was later sister. She has since been sister at Manchester Royal Eye Hospital.

### Manager's Notices.

**NOW READY, "The Hospital" Index to Vol. LXVII., Oct. 1919 to March 1920. Price 1s. 1d. per Copy post free.**

**All letters on business matters must be addressed to The Manager, "The Hospital," 28 and 29 Southampton Street, London, W.C.**

RATES FOR SUBSCRIPTION (payable in advance).

| | | |
|---|---|---|
| Three Months (including Postage) | ... ... | 3s. 3d. |
| Six Months do. | .. .. | 6s. 6d. |
| Twelve Months do. | ... .. | 13s. 0d. |

Should the cost of printing and paper, as well as increased postage, necessitate alteration of these rates, the period paid for will be reduced proportionately on unexpired subscriptions.

To ensure insertion, all advertisements for THE HOSPITAL must reach the Manager not later than Wednesday at noon in each case.

**It is especially requested that remittances be made by Cheque or Postal Order, and in halfpenny stamps only when the amount is under sixpence.**

*SUBSCRIPTION FORM.*

*Please send me* THE HOSPITAL *for* .............. *months, commencing with your issue of* ............................., *for which please find enclosed* ...................................

*Name* ...........................................

*Address* ...........................................

...........................................

...........................................

*Date* ...........................................

*To* ........................................... *Newsagent.*

**THE HOSPITAL**

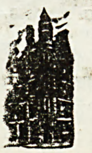

**The Modern Newspaper of Administrative Medicine and Institutional Life.**

**2nd October, 1920.** **COUPON**

